# 6-Shogaol Ameliorates Liver Inflammation and Fibrosis in Mice on a Methionine- and Choline-Deficient Diet by Inhibiting Oxidative Stress, Cell Death, and Endoplasmic Reticulum Stress

**DOI:** 10.3390/molecules29020419

**Published:** 2024-01-15

**Authors:** Ah Young Yang, Kiryeong Kim, Hyun Hee Kwon, Jaechan Leem, Jeong Eun Song

**Affiliations:** 1 Department of Immunology, School of Medicine, Daegu Catholic University, Daegu 42472, Republic of Korea; diddkdud123@naver.com (A.Y.Y.); kileyong93@naver.com (K.K.); 2 Department of Internal Medicine, School of Medicine, Daegu Catholic University, Daegu 42472, Republic of Korea; heeya0035@cu.ac.kr

**Keywords:** non-alcoholic steatohepatitis, 6-shogaol, inflammation, fibrosis

## Abstract

Non-alcoholic steatohepatitis (NASH) is becoming an increasingly serious global health threat, distinguished by hepatic lipid accumulation, inflammation, and fibrosis. There is a lack of approved pharmaceutical interventions for this disease, highlighting the urgent need for effective treatment. This study explores the hepatoprotective potential of 6-shogaol, a natural compound derived from ginger, in a methionine- and choline-deficient (MCD) dietary mouse model of NASH. Male C57BL/6J mice were subjected to the MCD diet for 4 weeks to induce NASH, with concurrent intraperitoneal administration of 6-shogaol (20 mg/kg) three times a week. While 6-shogaol did not impact body weight, liver weight, or hepatic lipid accumulation, it effectively mitigated liver injury, inflammation, and fibrosis in MCD diet-fed mice. Mechanistically, 6-shogaol inhibited lipid and DNA oxidation, restored hepatic glutathione levels, and regulated the expression of pro-oxidant and antioxidant enzymes. Furthermore, 6-shogaol inhibited apoptosis and necroptosis, as indicated by a decrease in TUNEL-stained cells and downregulation of apoptosis- and necroptosis-associated proteins. Additionally, 6-shogaol alleviated endoplasmic reticulum (ER) stress, as demonstrated by decreased expression of molecules associated with unfolded protein response pathways. These findings underscore the potential of 6-shogaol as a therapeutic intervention for NASH by targeting pathways related to oxidative stress, cell death, and ER stress.

## 1. Introduction

Non-alcoholic steatohepatitis (NASH) has evolved into a critical public health concern, with its global prevalence steadily increasing [1]. NASH is a severe manifestation of non-alcoholic fatty liver disease (NAFLD), characterized by hepatic lipid accumulation, along with inflammation and fibrosis. If left untreated, NASH has the potential to advance to cirrhosis, liver failure, and even liver cancer [2]. In spite of these grave outcomes, there are presently no approved drug therapies for NASH. This underscores the urgent necessity for extensive research aimed at developing effective therapies for NASH.

The pathogenesis of NASH revolves around a complex process in which several factors play pivotal roles, including oxidative stress, cell death, and endoplasmic reticulum (ER) stress [3]. It commences with hepatic lipid accumulation, setting the stage for subsequent events. Oxidative stress, stemming from an imbalance between the generation of reactive oxygen species (ROS) and the body’s antioxidant defenses, contributes to liver injury [4]. ROS damage cellular components and trigger inflammation. Cell death is another critical aspect of NASH pathogenesis, encompassing both apoptosis and necrosis [5]. As hepatocytes die, it fuels the inflammatory response, promoting disease progression. The accumulation of excessive misfolded or unfolded proteins can induce ER stress, prompting the unfolded protein response (UPR) to restore homeostasis [6]. This activation of UPR initiates inflammatory pathways and may result in hepatocyte dysfunction, thereby worsening NASH. These processes interplay and form a complex web in NASH pathogenesis, making them crucial focal points for research aimed at understanding and effectively treating this condition.

6-Shogaol is a naturally occurring compound found in ginger (*Zingiber officinale Roscoe*), characterized by its distinct ginger aroma and pungent taste [7]. It is considered one of the major bioactive constituents of ginger, well known for its diverse range of biological activities, including antioxidant, anti-inflammatory, anticancer, and neuroprotective properties [8,9]. In a recent study, we reported the protective effects of 6-shogaol against cisplatin-induced nephrotoxicity [10]. These protective effects were associated with the suppression of oxidative stress, cell death, and inflammation. Earlier studies have demonstrated the protective potential of 6-shogaol in various liver diseases such as acetaminophen-induced hepatotoxicity [11], sepsis-induced liver injury [12], and carbon tetrachloride-induced liver fibrosis [13] in mouse models. However, the potential effects of 6-shogaol on NASH have not been explored. To address this gap, we conducted an investigation to determine whether 6-shogaol exhibits hepatoprotective effects in NASH by employing a methionine- and choline-deficient (MCD) dietary mouse model. This model has been established as a reliable model for inducing NASH-like features, providing a platform to evaluate the impact of 6-shogaol on hepatic lipid accumulation, inflammation, and fibrosis [14,15]. Our focus extended beyond the observed pharmacological effects to the underlying molecular mechanisms contributing to these outcomes, specifically targeting oxidative stress, cell death, and ER stress.

## 2. Results

### 2.1. 6-Shogaol Does Not Change Body Weight, Liver Weight, and Hepatic Lipid Accumulation in MCD Diet-Fed Mice

**Figure 1:**
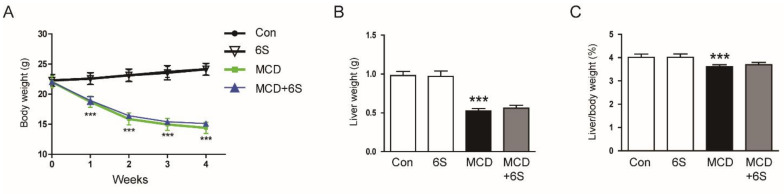

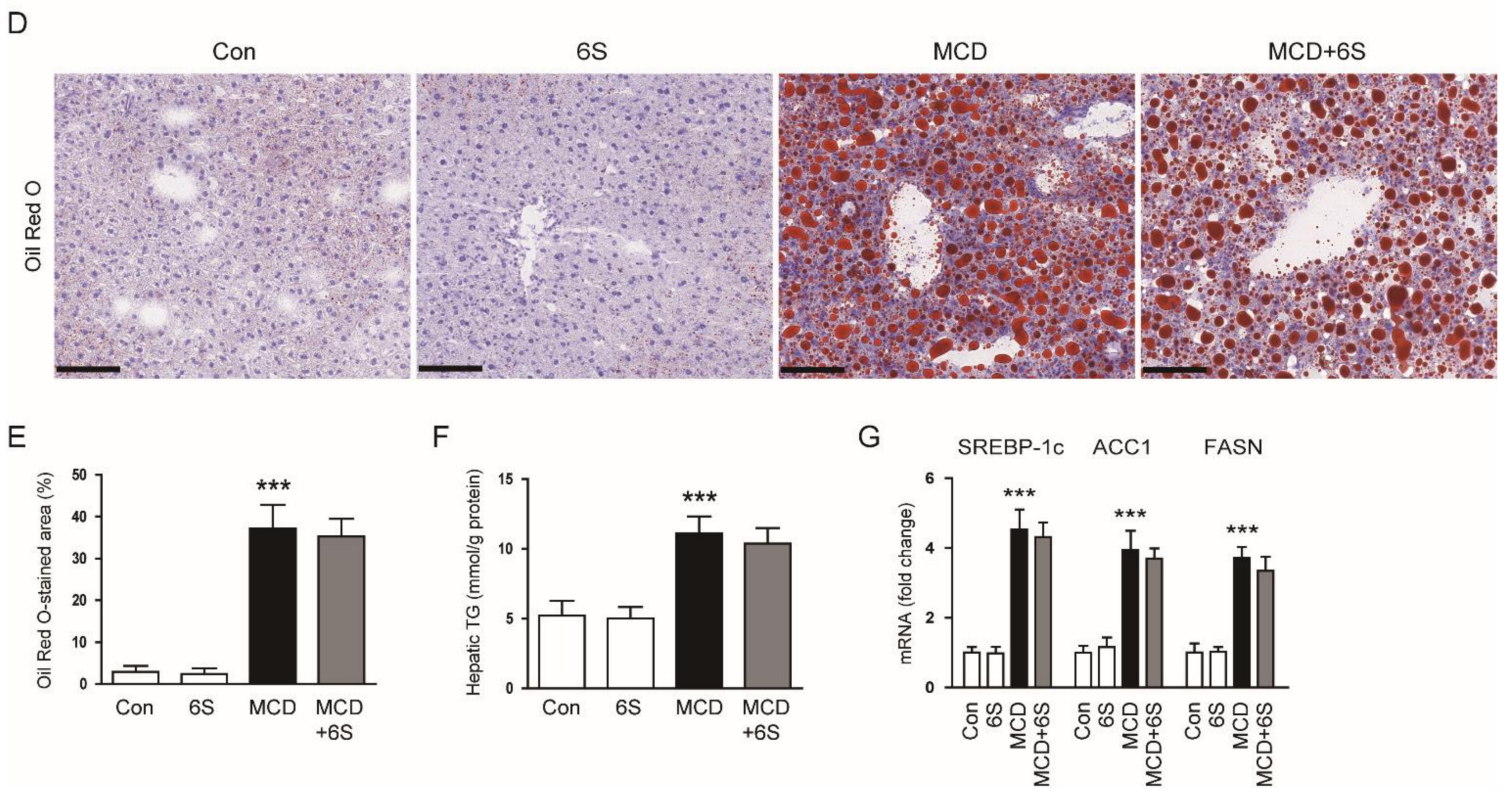
Effect of 6-shogaol on body weight, liver weight, and hepatic lipid accumulation in MCD-diet-fed mice. (**A**) Changes in mouse body weight over the feeding period. (**B**) Measurement of liver weight. (**C**) The liver-to-body weight ratio. (**D**) Oil red O staining of liver tissues. Scale bar = 100 μm. (**E**) Quantification of oil red O-stained areas. (**F**) Measurement of hepatic TG content. (**G**) mRNA expression of SREBP-1c, ACC1, and FASN in liver tissues. *n* = 8 per group. *** *p* < 0.001 vs. the Con group.

To assess the impact of 6-shogaol on NASH, we exposed C57BL/6J mice to the MCD diet for four weeks. Throughout this period, 6-shogaol was administered at a dose of 20 mg/kg via intraperitoneal injections three times a week. The MCD diet resulted in a significant reduction in body weight, liver weight, and the liver to body weight ratio (*p* < 0.001 for all; Figure 1A–C). Notably, 6-shogaol did not induce significant alterations in these parameters in mice subjected to the MCD diet (Figure 1A–C). As previously documented [16], liver tissue staining with oil red O revealed a substantial accumulation of triglycerides (TGs) in mice subjected to the MCD diet (*p* < 0.001; Figure 1D,E). A biochemical analysis of hepatic TG content confirmed the presence of MCD-diet-induced steatosis (*p* < 0.001; Figure 1F). Furthermore, mRNA expression of genes related to lipogenesis, such as sterol regulatory element binding protein-1c (SREBP-1c), acetyl-coenzyme A carboxylase 1 (ACC1), and fatty acid synthase (FASN), was upregulated after MCD diet feeding (*p* < 0.001 for all; Figure 1G). Importantly, 6-shogaol did not significantly affect these changes (Figure 1D–G).

### 2.2. 6-Shogaol Mitigates Liver Injury and Inflammation in MCD-Diet-Fed Mice

**Figure 2:**
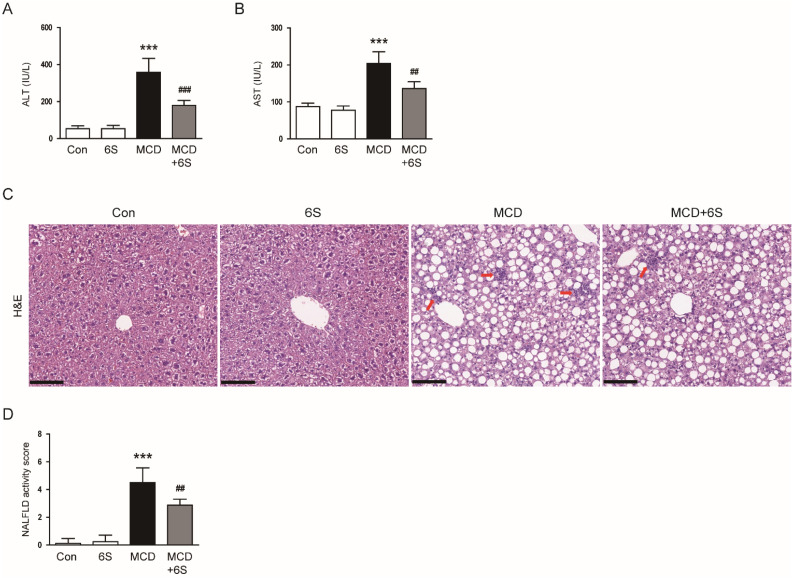
Mitigation of hepatic damage by 6-shogaol in MCD-diet-fed mice. (**A**) Serum ALT levels. (**B**) Serum AST levels. (**C**) H&E staining of liver tissues. Infiltration of inflammatory cells is indicated by the red arrows. Scale bar = 100 μm. (**D**) Evaluation of NAFLD activity scores. *n* = 8 per group. *** *p* < 0.001 vs. the Con group. ^##^ *p* < 0.01 and ^###^ *p* < 0.001 vs. the MCD group.

To evaluate liver injury severity, we measured the levels of alanine aminotransferase (ALT) and aspartate aminotransferase (AST) in the serum [17,18]. The MCD diet led to a significant increase in these markers, indicating substantial liver injury (*p* < 0.001 for both; Figure 2A,B). However, the administration of 6-shogaol effectively ameliorated the liver damage invoked by the MCD diet (*p* < 0.001 for ALT, *p* < 0.01 for AST; Figure 2A,B). Examination of liver tissues using hematoxylin and eosin (H&E) staining revealed the typical histological characteristics of NASH in MCD-diet-fed mice, including macrovesicular steatosis, inflammatory cell infiltration, and hepatocellular ballooning (Figure 2C). Notably, these changes were markedly reversed by 6-shogaol (Figure 2C). Moreover, 6-shogaol significantly alleviated the increase in the NAFLD activity score in mice exposed to the MCD diet (*p* < 0.01; Figure 2D).

**Figure 3:**
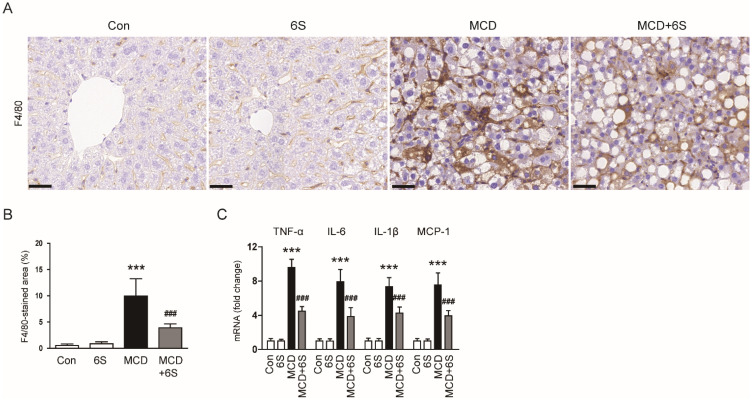
Diminishment of inflammation by 6-shogaol in MCD-diet-fed mice. (**A**) IHC staining of liver tissues for F4/80. Scale bar = 40 μm. (**B**) Percentage of areas displaying F4/80 staining. (**C**) mRNA expression of TNF-α, IL-6, IL-1β, and MCP-1 in liver tissues. *n* = 8 per group. *** *p* < 0.001 vs. the Con group. ^###^ *p* < 0.001 vs. the MCD group.

In addition to the accumulation of harmful lipids, inflammation is a prominent characteristic of NASH [3]. We subsequently examined the impact of 6-shogaol on liver inflammation. IHC staining using the pan-macrophage marker F4/80 [19] revealed a substantial reduction in the area of F4/80 staining in mice exposed to the MCD diet and treated with 6-shogaol (*p* < 0.001; Figure 3A,B). Furthermore, a quantitative real-time polymerase chain reaction (qPCR) analysis demonstrated a remarkable decrease in mRNA expression levels of key inflammatory mediators, including tumor necrosis factor-α (TNF-α), interleukin-6 (IL-6), IL-1β, and monocyte chemoattractant protein-1 (MCP-1), following treatment with 6-shogaol (*p* < 0.001 for all; Figure 3C).

### 2.3. 6-Shogaol Mitigates Liver Fibrosis in MCD-Diet-Fed Mice

**Figure 4:**
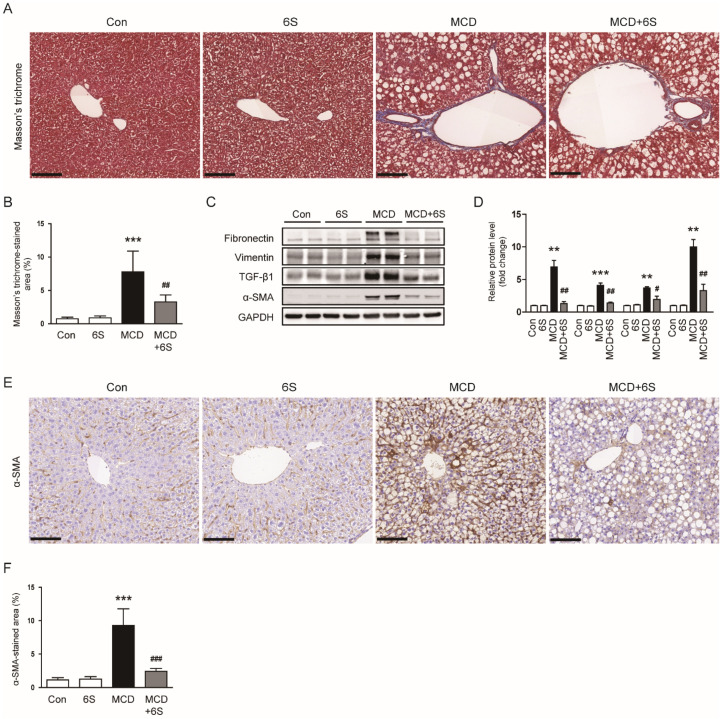
Mitigation of liver fibrosis by 6-shogaol in MCD-diet-fed mice. (**A**) Masson’s trichrome staining of liver tissues. Scale bar = 100 μm. (**B**) Percentage of areas exhibiting Masson’s trichrome staining. (**C**) Western blots displaying fibronectin, vimentin, TGF-β1, and α-SMA in liver tissues. (**D**) Quantitative analysis of Western blot results. (**E**) IHC staining of liver tissues for α-SMA. Scale bar = 100 μm. (**F**) Percentage of areas displaying α-SMA staining. *n* = 8 per group. ** *p* < 0.01 and *** *p* < 0.001 vs. the Con group. ^#^ *p* < 0.05, ^##^ *p* < 0.01, and ^###^ *p* < 0.001 vs. the MCD group.

Masson’s trichrome staining of liver specimens revealed a noticeable reduction in the area showing positive collagen fiber staining in mice exposed to the MCD diet and subsequently treated with 6-shogaol (*p* < 0.01; Figure 4A,B). Furthermore, elevated levels of fibronectin, vimentin, and transforming growth factor-β1 (TGF-β1) proteins in mice subjected to the MCD diet were remarkably decreased following treatment with 6-shogaol (*p* < 0.01 for fibronectin, *p* < 0.01 for vimentin, *p* < 0.05 for TGF-β1; Figure 4C,D). Additionally, 6-shogaol treatment downregulated the protein expression of α-smooth muscle actin (α-SMA), a widely recognized marker for myofibroblasts [20,21], in mice exposed to the MCD diet (*p* < 0.01; Figure 4C,D). IHC staining further validated the inhibitory action of 6-shogaol on α-SMA expression (*p* < 0.001; Figure 4E,F).

### 2.4. 6-Shogaol Reduces Oxidative Stress in MCD-Diet-Fed Mice 

**Figure 5:**
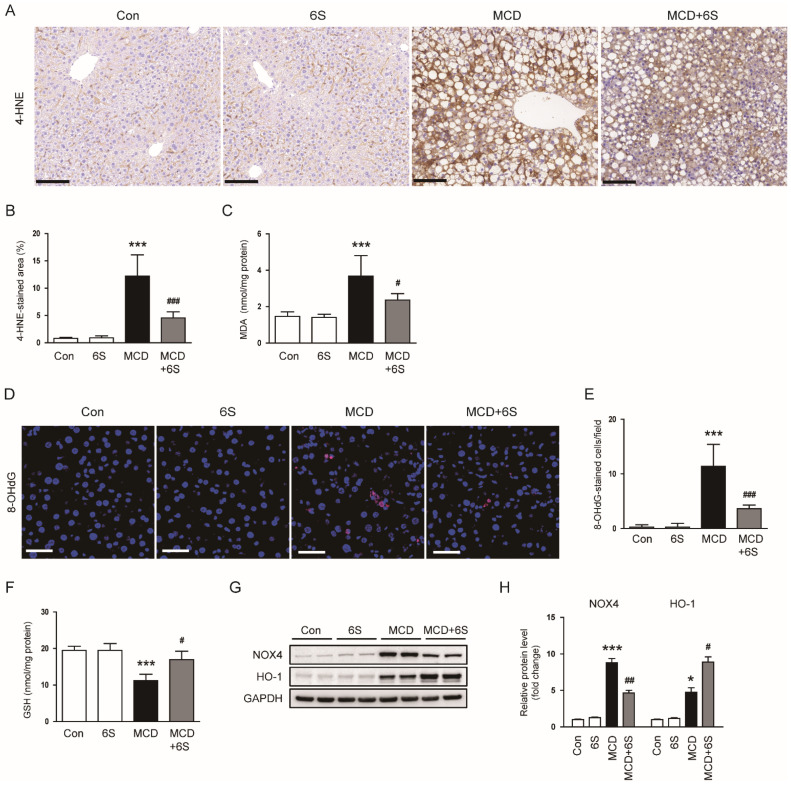
Attenuation of oxidative stress by 6-shogaol in MCD-diet-fed mice. (**A**) IHC staining of liver tissues for 4-HNE. Scale bar = 100 μm. (**B**) Percentage of areas displaying 4-HNE staining. (**C**) Hepatic MDA levels. (**D**) IF staining of liver tissues for 8-OHdG. Nuclei were counterstained with DAPI. Scale bar = 50 μm. (**E**) Number of 8-OHdG-stained cells. (**F**) Hepatic GSH levels. (**G**) Western blots displaying NOX4 and HO-1 in liver tissues. (**H**) Quantitative analysis of Western blot results. *n* = 8 per group. * *p* < 0.05 and *** *p* < 0.001 vs. the Con group. ^#^ *p* < 0.05, ^##^ *p* < 0.01, and ^###^ *p* < 0.001 vs. the MCD group.

Given the well-established critical role of oxidative stress in NASH pathogenesis [4], we conducted an investigation into the impact of 6-shogaol on oxidative stress to elucidate the mechanisms responsible for its protective effects against NASH. IHC staining of 4-hydroxynonenal (4-HNE), a recognized biomarker for lipid oxidative damage [22], revealed elevated hepatic expression of 4-HNE in MCD-diet-fed mice (*p* < 0.001; Figure 5A,B). Notably, the administration of 6-shogaol markedly reduced 4-HNE expression in mice exposed to the MCD diet (*p* < 0.001; Figure 5A,B). Moreover, hepatic levels of malondialdehyde (MDA), another biomarker for lipid oxidation [23], were also significantly reduced by 6-shogaol (*p* < 0.05; Figure 5C). Immunofluorescence (IF) staining for 8-hydroxy-2′-deoxyguanosine (8-OHdG), a marker for oxidative DNA damage [24], showed that the increased number of 8-OHdG-stained cells in mice exposed to the MCD diet was largely mitigated by 6-shogaol (*p* < 0.001; Figure 5D,E). Additionally, 6-shogaol effectively restored the reduced hepatic glutathione (GSH) levels in mice exposed to the MCD diet (*p* < 0.05; Figure 5F). Furthermore, MCD diet feeding resulted in an elevation of protein levels of the pro-oxidant enzyme NADPH oxidase 4 (NOX4) and the antioxidant enzyme heme oxygenase-1 (HO-1) in the liver (*p* < 0.001 for NOX4, *p* < 0.05 for HO-1; Figure 5G,H). Importantly, 6-shogaol significantly inhibited the upregulation of NOX4 protein and enhanced HO-1 expression in mice exposed to the MCD diet (*p* < 0.01 for NOX4, *p* < 0.05 for HO-1; Figure 5G,H).

### 2.5. 6-Shogaol Inhibits Apoptosis and Necroptosis in MCD-Diet-Fed Mice 

**Figure 6:**
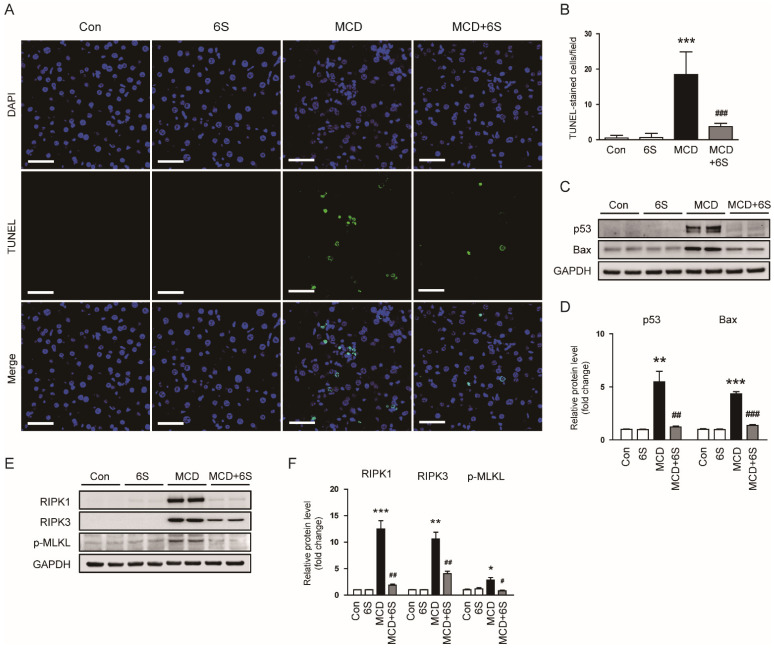
Inhibition of apoptosis and necroptosis by 6-shogaol in MCD-diet-fed mice. (**A**) TUNEL assay on liver tissues. Nuclei were counterstained with DAPI. Scale bar = 50 μm. (**B**) Number of TUNEL-stained cells. (**C**) Western blots displaying p53 and Bax expression in liver tissues. (**D**) Quantitative analysis of Western blot results. (**E**) Western blots displaying RIPK1, RIPK3, and p-MLKL expression in liver tissues. (**F**) Quantitative analysis of Western blot results. *n* = 8 per group. * *p* < 0.05, ** *p* < 0.01, and *** *p* < 0.001 vs. the Con group. ^#^ *p* < 0.05, ^##^ *p* < 0.01, and ^###^ *p* < 0.001 vs. the MCD group.

Hepatocyte death due to the abnormal accumulation of lipids plays a pivotal role in the development of inflammation and fibrosis in NASH [5]. Emerging evidence suggests that apoptosis and necroptosis are intricately involved in NASH pathogenesis [5,25]. To assess the protective impact of 6-shogaol on hepatocyte apoptosis, a terminal deoxynucleotidyl transferase dUTP nick end labeling (TUNEL) assay was conducted on liver sections. The MCD diet led to a significant increase in the number of TUNEL-stained cells (*p* < 0.001; Figure 6A,B). Importantly, 6-shogaol remarkably suppressed hepatocyte apoptosis in MCD-diet-fed mice (*p* < 0.001; Figure 6A,B). Additionally, elevated protein levels of p53 and Bax in mice exposed to the MCD diet were notably reduced by 6-shogaol (*p* < 0.01 for p53, *p* < 0.001 for Bax; Figure 6C,D). Furthermore, we assessed the effect of 6-shogaol on necroptosis by evaluating the protein levels of receptor-interacting serine/threonine-protein kinase 1 (RIPK1), RIPK3, and p-mixed lineage kinase domain-like protein (p-MLKL) in the liver. 6-Shogaol significantly downregulated the expression of these necroptosis-associated proteins in MCD-diet-fed mice (*p* < 0.01 for RIPK1, *p* < 0.01 for RIPK3, *p* < 0.05 for p-MLKL; Figure 6E,F). Collectively, these results indicate that 6-shogaol effectively mitigates apoptosis and necroptosis in mice exposed to the MCD diet.

### 2.6. 6-Shogaol Suppresses ER Stress in MCD-Diet-Fed Mice 

**Figure 7:**
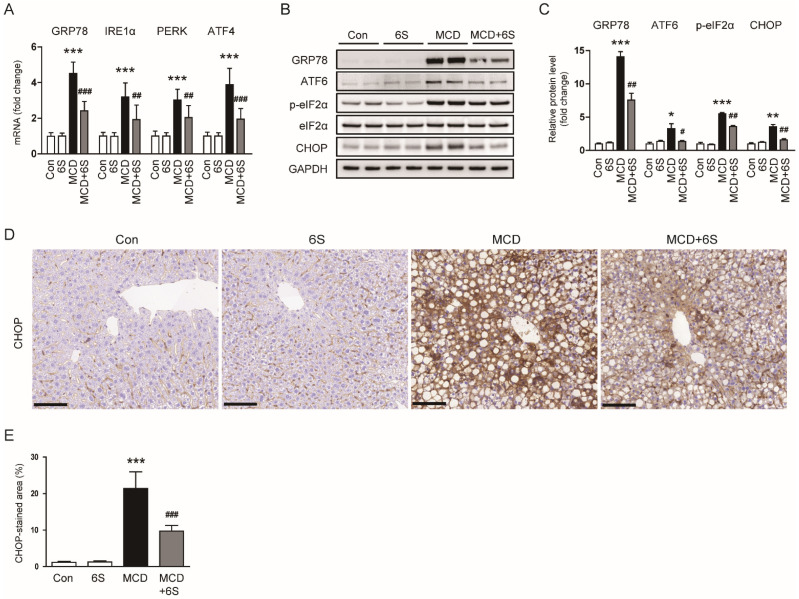
Suppression of ER stress by 6-shogaol in MCD-diet-fed mice. (**A**) Evaluation of mRNA expression of GRP78, IRE1α, PERK, and ATF4 in liver tissues. (**B**) Western blots depicting GRP78, ATF6, p-eIF2α, and CHOP in liver tissues. (**C**) Quantitative analysis of Western blot data. (**D**) IHC staining of liver tissues for CHOP. Scale bar = 100 μm. (**E**) Percentage of areas displaying CHOP staining. *n* = 8 per group. * *p* < 0.05, ** *p* < 0.01, and *** *p* < 0.001 vs. the Con group. ^#^ *p* < 0.05, ^##^ *p* < 0.01, and ^###^ *p* < 0.001 vs. the MCD group.

Accumulating evidence underscores the substantial role of ER stress in the development and progression of NASH [6,26]. Therefore, we next examined the impact of 6-shogaol on ER stress by assessing the expression of ER stress markers. Mice fed the MCD diet exhibited elevated mRNA levels of glucose-regulated protein 78 (GRP78), inositol-requiring enzyme 1α (IRE1α), protein kinase RNA-like ER kinase (PERK), and activating transcription factor 4 (ATF4) (*p* < 0.001 for all; Figure 7A). However, the expression of these markers was significantly reduced by 6-shogaol (*p* < 0.001 for GRP78, *p* < 0.01 for IRE1α, *p* < 0.01 for PERK, *p* < 0.001 for ATF4; Figure 7A). Additionally, 6-shogaol remarkably decreased the elevated levels of GRP78, ATF6, p-eukaryotic initiation factor 2α (p-eIF2α), and CCAAT/enhancer-binding protein homologous protein (CHOP) proteins in MCD-diet-fed mice (*p* < 0.01 for GRP78, *p* < 0.05 for ATF6, *p* < 0.01 for p-eIF2α, *p* < 0.01 for CHOP; Figure 7B,C). The inhibitory action of 6-shogaol on CHOP expression was further confirmed using IHC staining (*p* < 0.001; Figure 7D,E). Altogether, 6-shogaol effectively suppresses ER stress in mice exposed to the MCD diet.

## 3. Discussion

The clinical significance of NASH lies in its potential to progress to more severe liver diseases, such as cirrhosis and liver cancer, making it a pressing public health concern [27]. The urgent need for NASH therapy development is driven by the absence of approved treatments for this condition, which poses a significant health and economic burden worldwide. The present study demonstrated that 6-shogaol has protective effects against liver inflammation and fibrosis in NASH. These effects are associated with suppression of oxidative stress, cell death (apoptosis and necroptosis), and ER stress.

Utilizing natural compounds for NASH treatment is crucial, offering distinct advantages. Derived from plant sources with a rich history in traditional medicine, these compounds are known for their tolerability and low toxicity across diverse cultures [28,29]. Additionally, they often exhibit multifaceted mechanisms of action targeting multiple pathways associated with NASH pathology [30]. 6-Shogaol, a bioactive compound isolated from ginger, holds promise as a potential therapeutic option for various diseases, given its potent pharmacological activities, including antioxidant and anti-inflammatory effects [8,9]. The present study investigated the impact of 6-shogaol on NASH using an MCD dietary mouse model. This model offers the advantage of inducing rapid and severe hepatic steatosis, inflammation, and fibrosis [14]. However, a notable drawback is that it can induce significant weight loss, a feature not typically observed in NASH patients [31]. In line with prior studies [32,33], our data revealed that mice exposed to the MCD diet for 4 weeks displayed hepatic steatosis, inflammation, and fibrosis, along with a substantial reduction in body weight and liver weight. Treatment with 6-shogaol significantly alleviated NASH induced by the MCD diet, while showing no discernible impact on changes in body weight and liver weight. It is noteworthy that hepatic lipid accumulation remained unchanged despite the administration of 6-shogaol. SREBP-1c is a key transcription factor that promotes hepatic lipogenesis in NASH by upregulating the expression of lipogenic genes such as ACC1 and FASN [34]. A previous study documented the inhibitory action of 6-shogaol on SREBP-1 levels in human colorectal cancer cells [35]. However, our data showed that 6-shogaol did not alter the mRNA expression of lipogenic genes in mice exposed to the MCD diet.

In NASH, inflammation plays a central role, and macrophages are key contributors to this process [36]. In our study, feeding mice the MCD diet led to a substantial accumulation of macrophages and an excessive production of cytokines within the liver. Nevertheless, the administration of 6-shogaol remarkably inhibited both the macrophage accumulation and cytokine production in mice exposed to the MCD diet. Macrophages within the liver respond to inflammatory signals and promote the secretion of cytokines, exacerbating liver inflammation [36]. These pro-inflammatory cytokines further stimulate the recruitment and activation of additional immune cells, perpetuating a cycle of inflammation that can drive the progression of NASH [36]. In line with our results, Qiu et al. showed that 6-shogaol alleviated macrophage accumulation and cytokine production in a murine model of liver fibrosis induced by carbon tetrachloride [13]. Similarly, Kim et al. reported a significant reduction in both the number of macrophages and levels of cytokine expression in a mouse model of periodontitis upon treatment with 6-shogaol [37]. In the present study, the treatment with 6-shogaol was found to ameliorate fibrotic changes in mice exposed to the MCD diet, in addition to reducing inflammation. This was evidenced by a decrease in collagen fibers as observed in Masson’s trichrome staining and a reduction in the production of extracellular matrix proteins in the liver. Consistent with our results, recent studies have also demonstrated the anti-fibrotic effects of 6-shogaol in both the liver [13] and heart [38]. Furthermore, 6-shogaol remarkably inhibited the accumulation of α-SMA-positive myofibroblasts in mice exposed to the MCD diet. Myofibroblasts play a crucial role in fibrosis development in NASH, primarily responsible for the excessive production of extracellular matrix components, leading to the accumulation of fibrotic tissue in the liver [39]. These specialized cells become activated in response to liver injury and inflammation, and their persistent activation can lead to the progression of fibrosis, ultimately affecting liver function and structure [40].

Oxidative stress plays a pivotal role in the pathophysiology of NASH [4]. In NASH, the excessive accumulation of lipids in hepatocytes leads to an increase in ROS generation. These elevated levels of ROS can cause damage to cellular components, including DNA, lipids, and proteins. This oxidative damage triggers inflammation and contributes to the progression of NASH, leading to fibrosis and liver injury [4]. In this study, 6-shogaol exhibited a significant ability to inhibit oxidative stress in mice exposed to the MCD diet. This effect was substantiated by significant reductions in both lipid peroxidation and DNA oxidation along with the restoration of hepatic GSH levels. These findings underscore the effectiveness of 6-shogaol in alleviating the adverse impacts of oxidative stress induced by the MCD diet. Furthermore, our study unveiled that the MCD diet triggered the upregulation of NOX4 and HO-1 in mice. While NOX4, a pro-oxidant enzyme, contributes to ROS generation in various pathological processes [41,42], HO-1 operates as an antioxidant enzyme, safeguarding against oxidative damage and bolstering cellular resilience [43,44]. Therefore, the elevated expression of HO-1 in response to the MCD diet seems to represent a defensive reaction against oxidative damage. Importantly, 6-shogaol not only dampened NOX4 expression but also heightened HO-1 expression in mice exposed to the MCD diet. Consistent with these findings, our previous work demonstrated 6-shogaol’s ability to inhibit NOX4 expression in cisplatin-induced nephrotoxicity [10]. Additionally, several studies have shown the activation of the HO-1 pathway induced by 6-shogaol [45,46]. In essence, our data confirm that alleviating oxidative stress is one of the key strategies in the treatment of NASH, and 6-shogaol may emerge as a promising intervention candidate.

In the complex milieu of NASH pathogenesis, apoptosis and necroptosis are recognized as central players, exerting substantial impact on the progression of liver injury [5,25]. Apoptosis, a tightly regulated form of cell death, is triggered by various factors, including oxidative stress, lipotoxicity, and inflammatory signals. This form of cell death not only leads to a reduction in the number of functional hepatocytes but also fuels inflammation and fibrogenesis, thereby exacerbating liver injury [5]. Necroptosis, on the other hand, represents a form of regulated necrosis characterized by a cascade of distinct molecular events, particularly the RIPK1-RIPK3-MLKL axis, culminating in cell rupture and inflammation [47]. In NASH, necroptosis contributes to hepatocyte death, and the released cellular contents serve as danger signals, further amplifying the inflammatory response [25]. The interplay between apoptosis and necroptosis established a complex network of cell death pathways, collectively influencing the progression and severity of NASH [5,25]. Given the documented anti-apoptotic and anti-necroptotic effects of 6-shogaol [10,48], our study investigated its impact on apoptosis and necroptosis in MCD-diet-fed mice. As expected, mice exposed to the MCD diet exhibited increased apoptosis and necroptosis in the liver, as evidenced by reduced TUNEL-stained apoptotic cells and altered expression of apoptosis- and necroptosis-related molecules. However, 6-shogaol effectively inhibited both processes in mice exposed to the MCD diet. These findings imply that the anti-apoptotic and anti-necroptotic effects of 6-shogaol may contribute to its beneficial actions on MCD-diet-induced NASH.

ER stress is a cellular condition that arises when the folding capacity of the ER is overwhelmed, leading to the accumulation of misfolded or unfolded proteins [26]. In response to this disturbance, cells activate UPR pathways to restore ER homeostasis. The UPR comprises several adaptive mechanisms aimed at reducing the protein-folding load, degrading misfolded proteins, and enhancing the overall folding capacity of the ER [26]. In the context of NASH pathogenesis, ER stress has emerged as a critical player [6,26]. Our study demonstrated the inhibitory action of 6-shogaol on ER stress in mice exposed to the MCD diet. In NASH, excessive accumulation of lipids in hepatocytes contributes to an increased demand for protein synthesis, overwhelming the ER’s folding capacity and triggering ER stress [6,26]. The UPR is then activated to mitigate this stress, but persistent or unresolved ER stress can lead to cell dysfunction and death. ER stress in NASH is associated with several pathological consequences. Firstly, it exacerbates oxidative stress by promoting the generation of ROS. IRE1α, a key mediator of the UPR, has been implicated in promoting oxidative stress during ER stress [49]. IRE1α is a ubiquitously expressed enzyme endowed with endoribonuclease (RNase) and serine/threonine protein kinase functions. Upon activation, IRE1α can initiate signaling cascades that contribute to the generation of ROS [49]. Furthermore, ER stress disrupts cellular calcium homeostasis, leading to an increase in cytosolic calcium levels. Elevated levels of calcium can stimulate the activation of ROS-producing entities such as NOX enzymes, a significant contributor to ROS generation [50]. Secondly, ER stress contributes to inflammation by activating inflammatory pathways. The UPR triggers inflammatory pathways, including the activation of NF-κB and the generation of cytokines, contributing to the inflammatory response associated with ER stress [51,52]. For instance, Willy et al. demonstrated the critical role of CHOP in linking ER stress to NF-κB activation in NASH pathogenesis [53]. Additionally, sustained ER stress can induce hepatocyte death, further amplifying liver injury and fibrosis in NASH [6]. IRE1α RNase activity orchestrates the unconventional splicing of X-box binding protein 1 (XBP1) mRNA, resulting in the generation of the transcription factor sXBP1 [49]. This process enhances ER protein folding, initially fostering an adaptive UPR. However, under pathological ER stress conditions, sXBP1 from IRE1α activation transactivates a set of pro-apoptotic UPR-related genes, including CHOP [54]. Additionally, IRE1α’s RNase activity is capable of degrading mRNAs and microRNAs through a regulated process known as IRE1α-dependent decay, thereby contributing to inflammation and cell death [55]. Overall, our findings suggest that 6-shogaol improves liver inflammation, fibrosis, oxidative stress, and hepatocyte death, possibly through the inhibition of ER stress.

## 4. Materials and Methods

### 4.1. Animal Experiments

**Figure 8:**
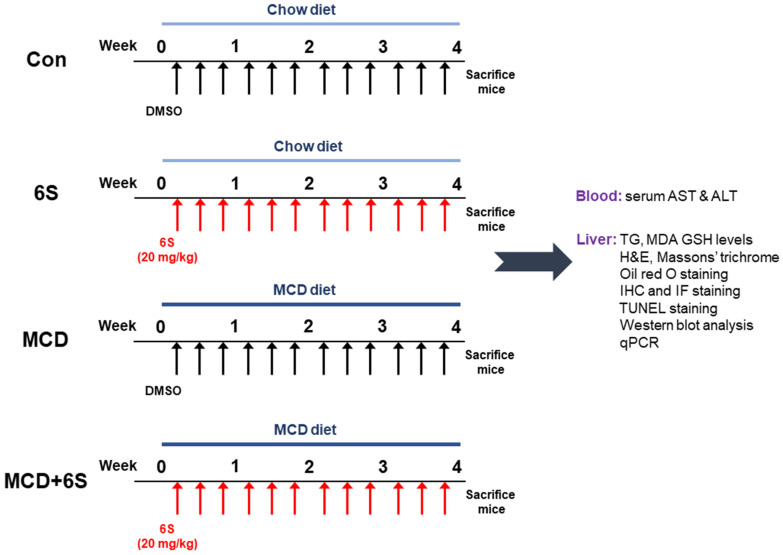
An overview of the experimental design and methodologies used in this study.

Six-week-old male C57BL/6J mice were obtained from HyoSung Science (Daegu, Republic of Korea) and acclimatized for one week in a controlled environment (20–24 °C, 12/12 h light/dark cycle) with free access to water and food. Subsequently, they were randomly assigned to four groups (*n* = 8 per group): Con, 6S, MCD, and MCD + 6S. The Con and 6S groups received a control diet with sufficient methionine and choline, while the MCD and MCD + 6S groups were fed an MCD diet for four weeks to induce NASH. These diets were procured from Dyets (Bethlehem, PA, USA) and their composition is detailed in prior literature [56]. The 6S and MCD + 6S groups received intraperitoneal injections of 6-shogaol (20 mg/kg; Sigma-Aldrich, St. Louis, MO, USA) three times a week concurrently with the MCD diet. To prepare the injection solution, 6-shogaol was first dissolved in dimethyl sulfoxide (DMSO) to make a stock solution. This stock solution was then diluted with phosphate-buffered saline (PBS) to attain the target concentration of 20 mg/kg for administration. The final injection solution, with a pH of 7.4, had a DMSO concentration of 2.4% (*v*/*v*). This specific concentration of DMSO was carefully chosen to ensure that 6-shogaol remained sufficiently soluble, while simultaneously minimizing any potential toxicity or irritation to the mice. The injection volume was adjusted based on each mouse’s body weight to ensure accurate dosing. Meanwhile, the Con and MCD groups received an equivalent volume of the vehicle during the same period. The dosage of 6-shogaol was determined based on established studies [10,45]. The body weight of each mouse was monitored weekly throughout the experiment. At the end of the experiment, all the mice were euthanized and blood samples were collected via cardiac puncture. At the same time, liver tissues were weighed and collected for a subsequent analysis. Our experimental design and methodologies are summarized in Figure 8. The Institutional Animal Care and Use Committee of Daegu Catholic University Medical Center approved our experimental protocol (DCIAFCR-220628-11-Y). 

### 4.2. Biochemical Analysis

Hepatic TG levels were measured using the triglyceride assay kit (Abcam, Cambridge, MA, USA). Serum ALT and AST levels were assessed using an automatic analyzer (Hitachi, Osaka, Japan). Hepatic MDA concentrations were determined using the MDA assay kit (Sigma-Aldrich, St. Louis, MO, USA), and hepatic GSH levels were measured with the GSH detection kit (Enzo Life Sciences, Farmingdale, NY, USA). All analyses followed the manufacturers’ recommended protocols.

### 4.3. H&E, Masson’s Trichrome, and Oil Red O Staining

The liver tissues were rapidly fixed in a 4% paraformaldehyde solution and subsequently embedded in paraffin. These paraffin-embedded tissue blocks were sectioned and mounted on glass slides. Staining with H&E or Masson’s trichrome was performed, and the slides were examined using the A1+ confocal microscope (Nikon, Tokyo, Japan). Liver injury severity was assessed based on the NAFLD activity score, a semi-quantitative measure categorizing each case for steatosis (0–3), lobular inflammation (0–3), or hepatocellular ballooning (0–2) [32,57]. Evaluations were conducted across five randomly selected fields (200× magnification) of H&E-stained sections for each sample. For oil red O staining, frozen liver sections were quickly fixed in 4% paraformaldehyde. Subsequently, the cytoplasmic lipid droplets were stained using an oil red O solution (Sigma-Aldrich, St. Louis, MO, USA), with nuclei counterstained with hematoxylin [16]. Quantification of the area stained with oil red O or Masson’s trichrome was performed using i-Solution DT software version 11.0 (IMT i-Solution, Coquitlam, BC, Canada) from five random fields (200× magnification for Masson’s trichrome and 400× magnification for oil red O) for each sample.

### 4.4. IHC and IF Staining

The liver sections were subjected to deparaffinization, rehydration, and immunostaining using antibodies targeting F4/80 (1:100; Cat. No. sc-377009; Santa Cruz Biotechnology, Dallas, TX, USA), α-SMA (1:100; Cat. No. A5228; Sigma-Aldrich, St. Louis, MO, USA), 4-HNE (1:100; Cat. No. ab48506; Abcam, Cambridge, MA, USA), or CHOP (1:100; Cat. No. MA1-250; Invitrogen, Carlsbad, CA, USA). After the washing step, the sections were incubated with an anti-mouse secondary antibody conjugated to horseradish peroxidase (1:100; Cat. No. 31431; Invitrogen, Carlsbad, CA, USA). Nuclei were counterstained with hematoxylin. Slides were examined using a slide scanner (3DHISTECH Pannoramic MIDI, Budapest, Hungary). The extent of staining for F4/80, α-SMA, 4-HNE, or CHOP was quantified in 5 random fields (400× magnification for F/480, 200× magnification for α-SMA, 4-HNE, and CHOP) for each sample. For IF staining, the liver sections were incubated with anti-8-OHdG antibodies (1:100; Cat. No. sc-66036; Santa Cruz Biotechnology, Dallas, TX, USA). Following washing, the sections were exposed to an anti-mouse secondary antibody labeled with Alexa Fluor 647 (1:100; Cat. No. A-21235; Invitrogen, Carlsbad, CA, USA). Nuclei were counterstained with DAPI. All slides were viewed under the A1+ confocal microscope (Nikon, Tokyo, Japan). The number of 8-OHdG-stained cells was counted in five random fields (600× magnification) for each sample.

### 4.5. TUNEL Staining

Apoptotic cells were identified using the TUNEL assay kit (Roche Diagnostics, Indianapolis, IN, USA) following the manufacturer′s guidelines. Nuclei were counterstained with DAPI, and the number of positive cells was counted in five random fields (600× magnification) for each sample.

### 4.6. Western Blot Analysis

Total protein extraction commenced with sample treatment using a lysis buffer (50 mM Tris-HCl, pH 7.4, 150 mM NaCl, 1% Triton X-100, 1% Na-deoxycholate, 0.1% SDS, 2 mM EDTA) containing a protease inhibitor cocktail (Sigma-Aldrich, St. Louis, MO, USA). Subsequently, these proteins were loaded onto polyacrylamide gels, transferred to nitrocellulose membranes, and then incubated overnight with primary antibodies targeting specific proteins, including fibronectin (1:1000; Cat. No. ab2413; Abcam, Cambridge, MA, USA), vimentin (1:1000; Cat. No. 3932; Cell Signaling Technology, Danvers, MA, USA), TGF-β1 (1:1000; Cat. No. MAB2402; R&D Systems, Minneapolis, MN, USA), α-SMA (1:1000; Cat. No. A5228; Sigma-Aldrich, St. Louis, MO, USA), NOX4 (1:1000; Cat. No. NB110-58849; Novus Biologicals, Littleton, CO, USA), HO-1 (1:1000; Cat. No. PA5-77833; Invitrogen, Carlsbad, CA, USA), p53 (1:1000; Cat. No. 2524; Cell Signaling Technology, Danvers, MA, USA), Bax (1:1000; Cat. No. sc-7480; Santa Cruz Biotechnology, Dallas, TX, USA), RIPK1 (1:1000; Cat. No. 4926; Cell Signaling Technology, Danvers, MA, USA), RIPK3 (1:1000; Cat. No. 95702; Cell Signaling Technology, Danvers, MA, USA), p-MLKL (1:1000; Cat. No. 37333; Cell Signaling Technology, Danvers, MA, USA), GRP78 (1:1000; Cat. No. 3177; Cell Signaling Technology, Danvers, MA, USA), ATF6 (1:1000; Cat. No. ab37149; Abcam, Cambridge, MA, USA), p-eIF2α (1:1000; Cat. No. 9721; Cell Signaling Technology, Danvers, MA, USA), eIF2α (1:1000; Cat. No. 9722; Cell Signaling Technology, Danvers, MA, USA), CHOP (1:1000; Cat. No. MA1-250; Invitrogen, Carlsbad, CA, USA), or glyceraldehyde-3-phosphate dehydrogenase (GAPDH; 1:3000; Cat. No. 5174; Cell Signaling Technology, Danvers, MA, USA). Antibodies were diluted in solutions of 5% skim milk. After incubating with primary antibodies, the membranes were washed and then probed with anti-mouse (1:1000; Cat. No. 7076; Cell Signaling Technology, Danvers, MA, USA) and anti-rabbit (1:1000; Cat. No. 7074; Cell Signaling Technology, Danvers, MA, USA) secondary antibodies for 2 h. Protein bands were visualized using enhanced chemiluminescence reagents (SuperSignal™ West Femto Maximum Sensitivity Substrate; Cat. No. 34096; Thermo Fisher Scientific, Waltham, MA, USA). The signal intensity was detected using the iBright CL1500 Imaging System (Thermo Fisher Scientific, Waltham, MA, USA) and quantified using ImageJ software version 1.53j (NIH, Bethesda, MA, USA).

### 4.7. qPCR

Total RNA was extracted from the samples utilizing the TRIzol reagent, followed by reverse transcription into cDNA using the PrimeScript RT Reagent Kit (TaKaRa, Tokyo, Japan). Subsequently, qPCR was performed using the Power SYBR Green PCR Master Mix (Thermo Fisher Scientific, Waltham, MA, USA) and specified primers detailed in Table 1. GAPDH served as an internal control.

### 4.8. Statistical Analysis

Data are presented as the mean ± standard deviation (SD). Differences between groups were analyzed using one-way or two-way ANOVA followed by Bonferroni’s post hoc tests. A *p* value < 0.05 was considered statistically significant.

## 5. Conclusions

In conclusion, our study reveals that 6-shogaol provides substantial protection against liver damage in a NASH mouse model. It effectively diminishes inflammation, fibrosis, oxidative stress, cell death, and ER stress, while not adversely affecting overall metabolic health. This research offers significant insights into the protective potential of 6-shogaol in NASH treatment and contributes to the understanding of natural compounds in managing this condition. Further investigation is needed to uncover the detailed molecular mechanisms behind the effects of 6-shogaol.

## Figures and Tables

**Table 1:** List of primers.

Gene	Primer Sequence (5′→3′)	Accession No.
SREBP-1c	F: ACGGAGCCATGGATTGCACA R: AAGGGTGCAGGTGTCACCTT	NM_001358314
ACC1	F: GAATCTCCTGGTGACAATGCTTATT R: GGTCTTGCTGAGTTGGGTTAGCT	NM_133360
FASN	F: CTGAGATCCCAGCACTTCTTGA R: GCCTCCGAAGCCAAATGAG	NM_007988
TNF-α	F: ACTTCGGGGTGATCGGTCCCC R: TGGTTTGCTACGACGTGGGCTAC	NM_013693
IL-6	F: TACCACTTCACAAGTCGGAGGC R: CTGCAAGTGCATCATCGTTGTTC	NM_031168
IL-1β	F: TGCAGCTGGAGAGTGTGGATCCC R: TGTGCTCTGCTTGTGAGGTGCTG	NM_008361
MCP-1	F: GGGCCTGCTGTTCACAGTT R: CCAGCCTACTCATTGGGAT	NM_011333
GRP78	F: TGGTATTCTCCGAGTGACAGC R: AGTCTTCAATGTCCGCATCC	NM_001163434
IRE1α	F: GCATCACCAAGTGGAAGTATC R: ACCATTGAGGGAGAGGCATAG	NM_023913
PERK	F: AGCACTCAGATGGAGAGAGTCAG R: GCTATGGGAGTTGTTGGACTGT	NM_004836
ATF4	F: GAGCTTCCTGAACAGCGAAGTG R: TGGCCACCTCCAGATAGTCATC	NM_009716
GAPDH	F: ACTCCACTCACGGCAAATTC R: TCTCCATGGTGGTGAAGACA	NM_001289726

## Data Availability

The data presented in this study are available in the article.
